# Shaping learning objectives for biomedical artificial intelligence: Student-centered insights into novel cell visualization technology

**DOI:** 10.1017/cts.2025.10107

**Published:** 2025-09-26

**Authors:** Rachel Emily Liu-Galvin, Nicholas Sherwin, Selin Kavak, Victoria Liwang, Samuel Border, Mishal Khan, Sanjay Jain, Pinaki Sarder, Yulia A. Levites Strekalova

**Affiliations:** 1 Department of Health Services Research, Management, and Policy, College of Public Health and Health Professions, University of Florida, Gainesville, FL, USA; 2 J. Crayton Pruitt Family Department of Biomedical Engineering, College of Engineering, University of Florida, Gainesville, FL, USA; 3 John T. Milliken Department of Medicine, Washington University, St Louis, MO, USA; 4 Department of Medicine, College of Medicine, University of Florida, Gainesville, FL, USA

**Keywords:** Artificial intelligence, medical, undergraduate, educational technology, learning

## Abstract

**Background:**

Artificial intelligence (AI) technology is rapidly entering biomedical research, and there is a need to assess and develop curricula that address trainees’ learning objectives and interests. Studies of biomedical workforce development show that the prospective engagement of students in formulating educational objectives and activities improves motivation and learning outcomes. This study aimed to explore the educational applications of a novel AI-powered technology in undergraduate education.

**Methods:**

A mixed-methods approach using elicitation interviews and cultural domain analysis was applied to identify the salience of ideas around the educational uses of Functional Unit State Identification & Navigation with Whole Slide Images (FUSION), an AI-powered cell-visualization technology. Interviews from 21 students were reduced to learning application statements and assessed for cultural salience and clustering for potential educational applications.

**Results:**

Saturation was reached after 11 interviews, and analysis resulted in eight clusters of 25 unique consensus-based statements. Students thought of the technology as a tool for cell analysis and measuring, but they also viewed applications for medical and K-12 education, public engagement, and note-keeping for technology research. Methodologically, our study demonstrates the potential of cultural consensus for learner-centered curriculum development.

**Conclusions:**

Our findings suggest that trainees perceive many educational uses of FUSION, including those that fit traditional biomedical research curricula and translational applications. Trainees should be engaged in co-design to support and guide technology translation for educational use.

## Introduction

As emerging artificial intelligence (AI) technology continues to drive healthcare and biomedical education forward, there lies a significant challenge to translate innovative advancements into practical tools that meet the needs of students with varying skill levels and backgrounds. Utilized effectively, AI has the power to transform medical and biomedical education, providing collaborative, tailored learning opportunities. However, educators may struggle to keep up with the rapid pace of technological development and how best to incorporate AI-powered technologies into undergraduate curricula.

Learning objectives are the anticipated knowledge and skills that students should obtain after a period of learning [1–3] and are distinct from learning outcomes, which are the observed knowledge and skills that students have obtained [17,18,20]. Engaging students in developing learning objectives can help increase awareness of connections between classroom-acquired knowledge and its real-world application when they become part of the biomedical workforce [4]. Thus, engaging students in developing learning objectives for AI technologies can enhance their competencies in using AI-powered applications and equip them with the necessary skills to navigate the AI-driven future of medicine. Additionally, students from different backgrounds, disciplines, and stages will demonstrate different learning motivations and competencies. Thus, participatory co-design efforts help ensure that curricula are tailored to their diverse needs.

Previous examples of the successful engagement of students in the development of learning objectives include the involvement of medical students in incorporating informatics learning objectives into medical school curricula [5] and the development of active learning components in biomedical engineering courses across laboratory, classroom, simulation-based clinical, and clinically immersive real-world settings. Student engagement in identifying learning outcomes was linked to enhanced learning and trainee perceptions of adaptive, experiential learning environments [4]. Engaging students in the development and articulation of learning objectives thus ensures that the learning objectives are designed to meet students’ self-perceived needs, helping them to achieve their learning outcomes and setting them up for success in the workforce.

### Objectives

This study aimed to engage undergraduate students in the development of learning objectives for Functional Unit State Identification & Navigation with Whole Slide Images (FUSION), a new AI-powered tissue visualization and analysis technology. Specifically, we sought to enhance student-centered learning by bridging the gap between FUSION’s AI-powered biomedical and technological potential and its implementation into educational environments. Through interviews with students, we expected to identify multiple perceived uses of FUSION. Using cultural domain analysis (CDA), we planned to organize these into clusters of unique statements, which would then inform the development of potential learning objectives. The study set out to answer the following research questions:

**RQ1:** What applications for an AI-powered tissue visualization and analysis technology are salient among undergraduate students?

**RQ2:** What are trainee-centered learning objectives for undergraduate training in an AI-powered tissue visualization and analysis technology?

## Materials and methods

### Context

This study explored the usability of a new AI-powered technology, FUSION, which was developed under the Human BioMolecular Atlas Program (HuBMAP) [10,11]. HuBMAP is funded by the Common Fund at the National Institutes of Health and includes researchers across multiple U.S. and international institutions. HuBMAP’s goal is to create a comprehensive spatial map of the human body at the single-cell level to advance understanding of how cells interact in the human body [10] To achieve this, HuBMAP is entering its production phase for an infrastructure capable of mapping functional tissue units across different organs [10]. Under this initiative, the HuBMAP Infrastructure, Visualization, and Engagement team implemented deep learning algorithms, high-performance computing, and spatially resolved molecular omics to create FUSION. Since 2021, HuBMAP has also offered internships for undergraduate students to support biomedical workforce development by creating a pipeline of emerging graduates familiar with HuBMAP tools, data, and processes.

FUSION is an innovative visualization tool for analyzing tissue samples that automates the tracing of structural boundaries within biopsies, enabling the precise measurements of the size, shape, color, and texture of functional tissue units [11,12]. These measurements correlate with gene expression data, allowing researchers to map molecular information to specific areas in a biopsy, bridging the gap between pathology and molecular biology [11,12]. FUSION integrates AI by executing automated segmentation algorithms to extract the boundaries and locations of different structures in histological tissue. For kidney sections, these include glomeruli, globally sclerotic glomeruli, tubules, arteries, and arterioles. Of these structures, tubules are the most numerous, as a single biopsy can contain upwards of 2,000 tubules of several different sub-types. Also included in FUSION, though maybe not traditionally thought of as AI, are a variety of clustering and dimensional reduction algorithms that are used on high-dimensional data to group similar samples under a single broad label. Specifically, FUSION implements various cell deconvolution methods wherein spatial transcriptomics measurements are aligned with a single-nucleus RNA-sequencing reference object and transformed to provide a relative “score” for approximately 72 different kidney cell sub-types. Furthermore, FUSION offers several different methods for expert users (and non-computational users) to generate annotations for different structures within slides or for entire slides. This information can then be used to train machine learning algorithms, which can be deployed as custom components in FUSION.

### Consensus-based needs assessment using cultural domain analysis

To assess FUSION’s potential curricular applications and identify salient ideas among biomedical and electrical engineering undergraduate students, we employed CDA, a cognitive anthropology method. CDA aims to “describe the contents, structure, and distribution of knowledge in organized spheres of experience, or cultural domains [13].” CDA is concerned with how people with a shared experience think and talk about it [14]. CDA’s primary purpose is to enable the systematic identification of shared knowledge [15], and a “cultural domain” refers to knowledge shared by a group. Identifying cultural domains involves eliciting responses from interviewees or asking respondents to list items that they associate with a domain of understanding in an unconstrained way, called “free-listing [6,8,9].” Researchers then look for shared patterns among respondents, indicating potentially culturally learned domains [9]. Cultural domains can also be understood as “categories,” for example, animals or illnesses, that are shared and agreed among individuals and are about “perceptions rather than preferences [6,7].” CDA’s mixed methods approach makes it especially well-suited for generating hypotheses in emerging areas, such as integrating new AI technologies in biomedical education settings. As technology continues to play an ever-increasing role in medical education, there is a growing need to understand students’ shared knowledge and perceptions toward these platforms. By assessing student perceptions, instructors can develop learner-centered, rather than technology-centered, curricula. This assessment can also help map a module using research technology, such as FUSION, into existing applications of knowledge that are familiar to the students. This, in turn, can support the development of skills that span across different technology platforms and address higher-level learning objectives.

### Participants

Participants were recruited through emails sent to the listservs of the University of Florida’s biomedical and electrical engineering departments. In addition, we invited undergraduate students who participated in previous summer research experiences hosted by the research team. The invitation email introduced the study’s objectives, emphasized the voluntary nature of participation, and provided details about the $25 incentive. Those who expressed interest were provided further instructions for scheduling an elicitation interview via Zoom. The inclusion of students from both a general convenience sample and those with prior summer research experience was intended to capture a broader range of student experiences, learning motivations, and competencies. Given that students from different backgrounds, disciplines, and stages of training demonstrate different competencies, including students with differing levels of past research experience, it supports the generalizability and real-world applicability of the learning objectives developed in this study.

### Procedure

Upon joining the interview session, participants were informed that the interview would be recorded for transcription and analysis and were asked to provide verbal consent before proceeding. Participants received a brief overview of the study’s purpose, procedures, confidentiality protocols, and their rights as participants. The participants were then presented with a short 4-minute video that introduced the capabilities and functionalities of FUSION, ensuring all participants had the same level of understanding of the platform [16,17].

Following the video presentation, participants engaged in an individual elicitation interview. Interviews were divided between two interviewers, who asked interviewees the same three open-ended questions detailed in Appendix A1 to main consistency. The open-ended questions were designed to gather the participants’ thoughts, opinions, and perceptions of FUSION for undergraduate biomedical training. Participants were asked each question with intentionally varied wording multiple times to encourage deep reflection on their perspectives and experiences. Examples of how the questions were reframed are also detailed in Appendix A1. Meaningful engagement was evaluated based on list length (number of unique items provided by interviewees) and interview duration. Subsequently, participants were asked to complete a brief, anonymous demographic survey asking participants about their. The survey collected data on the following participant characteristics: race, ethnicity, gender identity, age, education level, current major, and prior research involvement.

### Analysis

Following the completion of the 21 interviews, transcripts were generated and analyzed. Each transcript underwent a manual review to identify unique statements representing individual themes expressed by participants. Direct quotations from each transcript were extracted, standardized based on the recurrence and repetition of ideas [18], and organized into ordered lists based on the order in which the participants mentioned them [6]. To prepare the data for the analysis, each response was assigned a unique ID, and ideas listed by each respondent were organized into lists, maintaining the order in which they were mentioned. The data was reviewed repeatedly for the consistency of wording and normalized for capitalization and punctuation. Next, Free List Analysis under R Environment using Shiny (FLARES) [19], an open-source, cloud-based software was used to identify salient learning objectives through systematic normalization and subsequent quantitative analyses of elicited needs statements. FLARES was used to conduct quantitative analyses of the cultural salience of the unique statements identified from the interview transcripts. The following metrics were assessed: the frequency of mention (how many participants mentioned a particular item), the relative frequency of mention (the proportion of participants who mentioned the item), and Smith’s index (a measure of cultural salience calculated using both the frequency of mention and the rank order (how early on the item is listed by each participant). The Smith Index was calculated using the following formula:

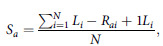




where *S*_*a*_ is the cultural salience of item *a; N* is number of lists (number of respondents), *L*_*i*_ is the length of list *i*, and *R*_*ai*_ is citation rank of item *a* in list *i* [8,20]. In the context of this study, cultural salience refers to the importance of the various use cases of FUSION as perceived by undergraduate students.

Following this, hierarchical cluster analysis was performed to generate a dendrogram illustrating how items were grouped into clusters.

## Results

Responses from 21 participants were analyzed. Table 1 reports the participants’ demographic makeup, academic background, and previous research experience.

**Table 1.** Demographics, academic background, and previous research experience of the participants

Demographic	Percent (%)	N
**Sex** Male Female	38.1 61.9	8 13
**Race** Asian Black or African American White Other More than one race Prefer not to answer	28.6 4.8 47.6 9.5 4.8 4.8	6 1 10 2 1 1
**Ethnicity** Hispanic or Latino Not Hispanic or Latino	47.6 52.4	10 11
**Age Group** 18 to 24 25 to 34	95.2 4.8	20 1
**Degree** Undergraduate	100	21
**Major** Biomedical Engineering Computer Science Electrical Engineering Business Law (Pre-Law Track) Nanomedicine	61.9 9.5 19.0 4.8 4.8	13 2 4 1 1
**Prior involvement in research** None / no involvement in research Some involvement in research Significant involvement in research	19.0 57.1 23.8	4 12 5
**If you have been involved in research, please select which areas of research you have been involved in. Select all that apply** [1]. Basic research Preclinical research Clinical research and clinical/behavioral trials Other[2]	47.6 33.3 4.8 28.6	10 7 1 6

^1^Percentages do not sum to 100 for this question since participants could select more than one response. ^2^Other responses were as follows: polymer synthesis (*n* = 1), electrical engineering sensor fusion research (*n* = 1), environmental science (*n* = 1), solid-state physics (*n* = 1), machine learning for electrical engineering (*n* = 1), and one unspecified (blank) response.

In total, 92 items were generated, of which there were 25 unique statements. The average list length, defined as the number of unique statements per individual, was 4.4. Saturation was achieved after 11 responses, at which point all 25 unique items had been cited. The data saturation plot is provided in Appendix A2. The average interview response length was 10 minutes and 52 s, the maximum was 28 minutes and 21 s, and the minimum was 6 minutes and 2 s. Table 2 reports the frequency of mention, the relative frequency of mention, and the Smith Index of the educational uses statements identified from the interview transcripts.

**Table 2.** Cultural saliency metrics for the perceived uses of cell visualization technology

Cited Items	Frequency of Mention	Relative Frequency of Mention	Smith Index
Analyzing cells/structures	14	0.667	0.463
Visualizing cells/structures	13	0.619	0.446
Detecting diseases/causes	10	0.476	0.315
Educating students	6	0.286	0.193
Sharing data	5	0.238	0.139
Learning about cells/structures	5	0.238	0.139
Counting cells/structures	5	0.238	0.124
Educating medical students	3	0.143	0.118
Identifying/differentiating features of cells/structures	3	0.143	0.098
Measuring cells/structures	3	0.143	0.084
Accessing data	3	0.143	0.043
Carrying out research	3	0.143	0.066
Collaborating with researchers	3	0.143	0.066
Communicating between patients and clinicians	3	0.143	0.062
Identifying abnormalities	2	0.095	0.052
Verifying information	2	0.095	0.041
Analyzing the effect of drugs on cells/structures	1	0.048	0.048
Educating elementary and high schoolers	1	0.048	0.048
Applying to medical malpractice	1	0.048	0.040
Imaging (radiologic/ultrasound)	1	0.048	0.032
Editing images	1	0.048	0.019
Troubleshooting a project	1	0.048	0.019
Educating the general public	1	0.048	0.016
Applying to genomic research	1	0.048	0.012
Keeping records	1	0.048	0.010

### Salience of the educational uses of technology

To address RQ1, we measured the cultural salience of the potential applications of FUSION among the undergraduate students we interviewed. Cultural salience refers to how prominent, important, or meaningful a particular item is among a specific group of individuals. An item is considered to be culturally salient if it is mentioned frequently across participants and tends to be mentioned early on by participants. Among the 25 unique statements provided by the 21 participants, the most frequently mentioned was: “analyzing cells/structures,” which was mentioned by 14 participants. Given that the data of 21 participants was analyzed, this result directly translated to a relative frequency of 0.667 for “analyzing cells/structures,” with two-thirds of participants mentioning this application. “Analyzing cells/structures” also had the highest Smith Index (0.463), indicating that this idea was the most salient within the students’ collective understanding of the potential uses of FUSION. The next most frequently cited statement was “visualizing cells/structures,” with 13 participants highlighting this use of FUSION during their interview, a relative frequency of mention of 0.619 (indicating that the idea was brought up by 61.9% of students interviewed), and a Smith Index of 0.446, indicating that this idea was also highly salient among the students interviewed. “Detecting diseases/causes” and “educating students” were also each cited multiple times, receiving 10 and 6 mentions, respectively, from the 21 participants, with relative frequencies of 0.476 and 0.286, and Smith Indices of 0.315 and 0.193, respectively. “Sharing data,” “learning about cells/structures,” and “counting cells/structures” were each mentioned 5 times, with Smith Indices of 0.139, 0.139, and 0.124, respectively. In contrast, nine statements were each mentioned only once, with the lowest Smith Index of 0.010 generated for the statement “keeping records.”

### Clusters of learning objectives

**Figure 1.**
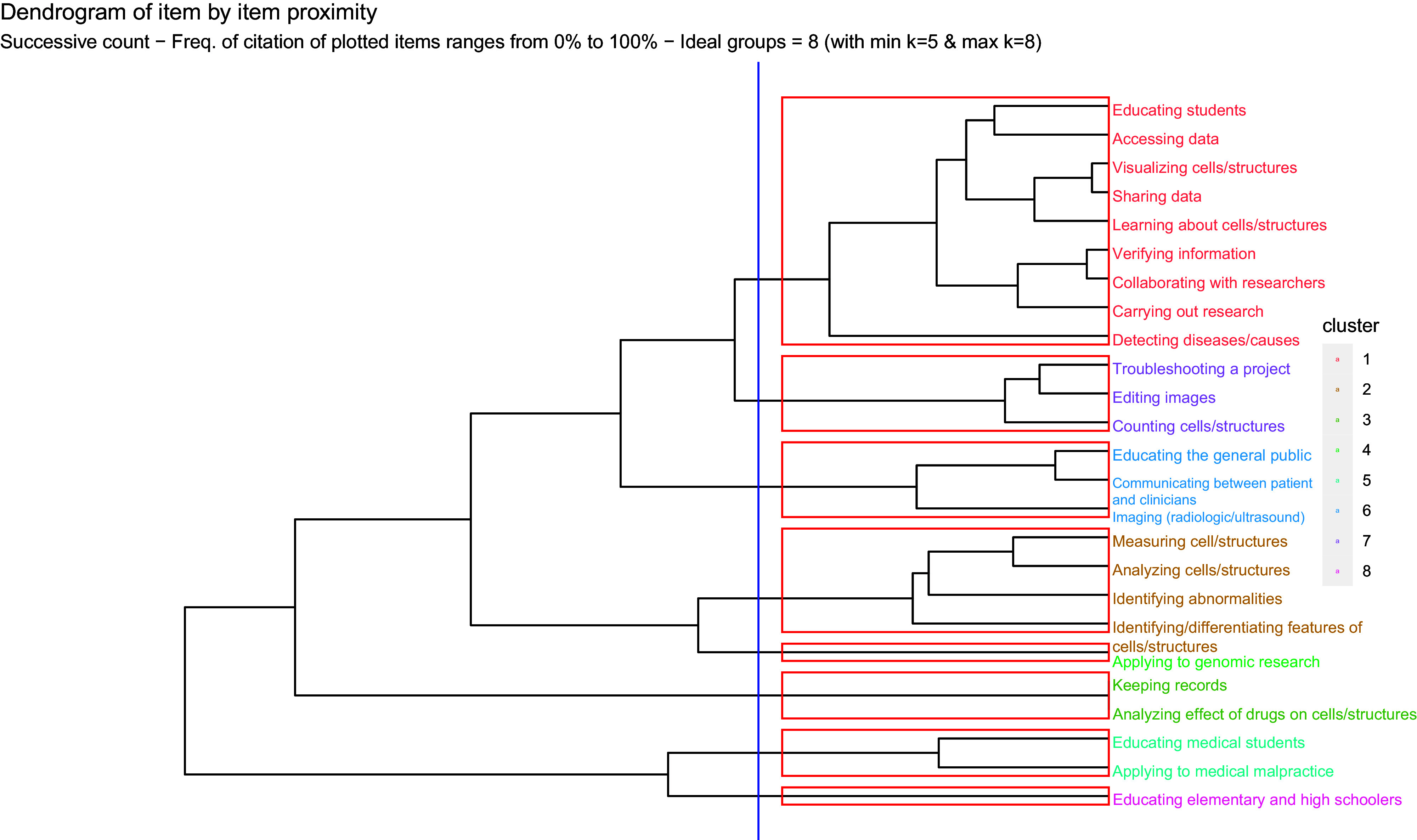
Dendrogram depicting item by item proximity for the cited items.

Using FLARES, a dendrogram was created to illustrate the item-by-item proximity for the 25 statements (Figure 1). This dendrogram demonstrates that the 25 items, or statements, fell into eight clusters, each containing at least one consensus item. In doing so, the hierarchical relationships between the statements can be evaluated, with each merge or split representing similarity or dissimilarity between each statement, respectively.

To address RQ2, we used these clusters as a framework to formulate potential learning objectives for FUSION. Table 3 presents a list of the items contained within each cluster, along with an interpretative label for each cluster, that is, a description of the common theme linking these items together, as well as a potential learning objective based on each cluster.

**Table 3.** Summary of educational use clusters and sample learning objectives

Interpretative Label	Cluster Items	Sample Learning Objective
**Cluster 1: Cell-level Technology in Higher Education**	Educating students Accessing data Carrying out research Collaborating with researchers Detecting diseases/causes Learning about cells/structures Sharing data Verifying information Visualizing cells/structures	Students will apply advanced technology to learn about cell structures and visualize cell structures.
**Cluster 2: Identification and Analysis in Cell, and Clinical Translational Research**	Analyzing cells/structures Identifying abnormalities Identifying/differentiating features of cells/structures Measuring cell/structures	Students will identify, measure, and analyze cell structures with the use of AI-powered technology.
**Cluster 3: Record-Keeping and Drug Evaluation**	Analyzing the effect of drugs on cells/structures Keeping records	Students will identify practices in laboratory record-keeping for technology-based research.
**Cluster 4: Genomic Research**	Applying to genomic research	Students will explore the use of cellular measurement technology for single-cell genomics research.
**Cluster 5: Medical Student Education**	Educating medical students Applying to medical malpractice	Students will explore tissues, cells, and cell structures with the use of advanced technology and discuss the potential applications of technology for clinical practice.
**Cluster 6: Public Education and Health Communication**	Communicating between patients and clinicians Educating the general public Imaging (radiologic/ ultrasound)	Students will be able to use FUSION to communicate scientific findings to the public and enhance patient-provider communication.
**Cluster 7: Image editing and troubleshooting**	Counting cells/structures Editing images Troubleshooting a project	Students will edit images of tissue samples and cells, and count cells and structures within cells.
**Cluster 8: Early STEM Education**	Educating elementary and high schoolers	Students will use advanced technology to present biological concepts visually and deliver accessible educational content to K-12 students.

## Discussion

This mixed-methods study investigated the perspectives of undergraduate students regarding the educational uses of a novel AI-powered tissue visualization and analysis technology, FUSION. Through learner-centered investigation, this study provided insights about potential applications of FUSION across a variety of educational, research, and clinical settings, and applied CDA to identify and formulate learning interests and objectives for students using this technology. Our analysis of the salience of unique statements revealed that many students thought that FUSION could be used for visualizing and analyzing cells and cell structures in the traditional context of higher education courses; these being the joint most frequently mentioned potential applications of FUSION in the student interviews. Our study also generated some potential applications of the software that were less salient, with nine unique statements receiving only one mention from the students we interviewed.

The learning objectives we formulated are applicable across multiple educational settings, including research laboratories, classrooms, and clinical settings. This finding is supported by previous studies that have shown that new educational tools can be implemented across various educational, research, and clinical settings [4]. As with Singh *et al* [4]., we also found that the process of engaging students in the development of learning objectives revealed that the students we interviewed possessed an awareness of the connections between classroom-acquired knowledge and opportunities to apply that knowledge in the real world and in cross-disciplinary learning environments. For example, students recognized that FUSION could be used both for educating students in the classroom as well as in real-world applications such as medicolegal cases involving medical malpractice.

The findings presented in this study align with previous research that has advocated for learner-centered approaches in curriculum development, further emphasizing the importance of tailoring educational content to student needs [21–25]. Many of the learning objectives we formulated were focused on specific applications of the FUSION software, such as visualizing and analyzing cells and cell structures and identifying abnormal features of cells. We used clusters generated in the CDA as a framework for formulating specific learning objectives tailored to address these specific learning outcomes. The clustering of learning objectives reflects the clusters generated through the hierarchical clustering analysis, as conducted using our CDA methodology. Each cluster represents a set of learning objectives that share similar cognitive characteristics, aligning with the study’s aim to identify and organize meaningful groupings of learning objectives. This clustering directly supports our goal of understanding how students conceptually relate different learning objectives together, a central focus of CDA. Our methodology, therefore, provides a demonstration of how learning objectives for new educational technologies can be developed and tailored to support individuals in achieving their learning needs. Tailoring learning objectives to the student is an approach that has demonstrated success in multiple studies, giving rise to empowering learning opportunities that also encourage collaboration with others [25–28].

Students also saw the versatility of FUSION use as an educational tool that could benefit learners from a spectrum of educational levels and backgrounds, ranging from elementary and high school students to medical students. Since some of the students interviewed mentioned that FUSION could be used for educating medical students, we formulated a learning objective that centered on implementing FUSION into a medical school curriculum as a tool to both educate medical students about tissues, cells, and cell structures and to deepen their understanding as to how an AI-powered software such as FUSION could be applied in their future practice as physicians. Some students also mentioned potential clinical applications of FUSION, such as to assist with communication between clinicians and patients, while another student mentioned that FUSION could be used in radiologic or ultrasound imaging; these examples further illustrate that students are cognizant of the connections between knowledge gained in the classroom and how that knowledge may be applied in the real world [4]. Our findings regarding the potential applications of FUSION both as an educational tool for medical students as well as in clinical practice are supported by previous studies that have emphasized the importance of incorporating such tools into medical education, in order to equip students with the skills they will need to thrive in the technology-driven future of medicine [21,29–32].

Methodologically, this study demonstrates the utility of CDA in a biomedical education context, where understanding shared knowledge structures can guide the creation of learner-centered educational objectives. The high cultural salience of specific functionalities (e.g., cell analysis and visualization) reveals a clear priority for these elements in educational design, highlighting their potential to be focal points in curriculum planning and instructional materials. Additionally, usability data obtained from the free-listing of applications enhances understanding of the user experience, informing iterative software design tailored to educational needs. These insights support the co-design of training modules that are not only technically accessible but also pedagogically relevant, encouraging engagement across varied skill levels. This study thus underscores the importance of integrating student feedback into the development of educational technology, fostering not only skill acquisition but also preparing trainees for the increasingly technology-driven landscape of biomedical research and healthcare.

### Limitations

This study offers valuable insights into the educational applications of cell-visualization AI technology from the student perspective; however, several limitations should be acknowledged. First, the use of a convenience sample drawn primarily from a single institution limits the generalizability of the findings. While this approach provided diverse perspectives within a controlled environment, a broader, multi-institutional sample would strengthen the validity of the results, particularly given potential differences in students’ backgrounds, prior knowledge, and exposure to similar technologies. Although the sample also included two students who had previously participated in a research experience at the Computational Microscopy Imaging Laboratory (CMIL), their limited prior exposure to the FUSION technology was unlikely to have significantly shaped their responses. Additionally, the inclusion of students with different research backgrounds was intended to enhance the generalizability of the findings.

Another important limitation was that participants were exposed to FUSION through a video demonstration rather than direct, hands-on interaction. This lack of hands-on experience likely limited their perceptions of FUSION’s usability, as their insights were shaped solely through passive observation. Additionally, while FUSION is an AI-based technology, deeper engagement with AI-specific components to make more robust claims about its educational value are needed. Finally, this study did not include a formal validation phase.

### Future research and validation of learning objectives

Future studies should provide students with hands-on experience using FUSION to directly assess its usability, accessibility, and integration within real-world educational settings, thereby enhancing ecological validity (that is, the degree to which the findings generalize to real-world educational contexts). This would yield valuable insights into FUSION’s usability, accessibility, and integration within active learning settings, potentially revealing challenges and benefits not evident in passive demonstrations. In addition to incorporating hands-on interaction with FUSION, future studies should also include explicit comparisons to expert-derived learning objectives to enhance the rigor of the findings and their applicability to formal curriculum development.

Since the study did not include a formal validation phase, further assessment is required, in which we plan to engage experts in medical education, AI, and curriculum design to review and confirm that the learning objectives derived from student interviews are relevant, comprehensive, and aligned with educational goals of students. We plan to conduct further interviews with instructors, faculty, AI experts, and curriculum designers to validate our findings, and integrate their expertise with the student perspectives obtained during this study. Following this validation, we aim to pilot-test the learning objectives in a small-scale educational setting using FUSION. This will allow us to assess whether the objectives are practical, measurable, and achievable, while also collecting feedback from both students and instructors to guide further refinement.

Based on our findings and these planned steps, we propose a user-informed iterative framework to inform both curriculum development and software optimization for FUSION. This type of framework has been successfully employed in other studies evaluating how AI-based educational technologies may be used to enhance students’ educational experiences [33]. It emphasizes a continuous feedback loop, beginning with user-informed learning objectives, followed by pilot implementation, evaluation, and iterative refinement. Through collaboration between educators, students, and software developers, this approach ensures that both pedagogical strategies and technological features evolve in alignment with learner needs and curricular standards.

## Conclusion

Advancements in educational technologies present both opportunities and challenges for biomedical science curriculum development. The development of learner-centered objectives can be supported by consensus-based approaches. Our findings provide valuable information into the perceptions and usability of AI-powered tissue visualization and analysis technology by revealing salient applications of such technology in the educational context among undergraduate students.

The AI-powered platform evaluated in this study, through elicitation interviews with potential users and a CDA approach, demonstrated significant potential to enhance undergraduate biomedical education. However, future research is needed, particularly that which provides students with direct hands-on experience using the technology, as well as a formal validation phase that incorporates instructor perspectives, to confirm and refine the learning outcomes developed.

## Supporting information

10.1017/cts.2025.10107.sm001 Liu-Galvin et al. supplementary material Liu-Galvin et al. supplementary material

## Supplementary material

The supplementary material for this article can be found at https://doi.org/10.1017/cts.2025.10107

## References

[1] Osueke B , Mekonnen B , Stanton JD. How undergraduate science students use learning objectives to study. J Microbiol Biol Educ. 2018;19:19.2.69. doi: 10.1128/jmbe.v19i2.1510. PMC6022773 29983848

[2] Harden RM. Learning outcomes and instructional objectives: is there a difference? Med Teach. 2002;24:151–155. doi: 10.1080/0142159022020687. 12098434

[3] Allan J. Learning outcomes in higher education. Stud High Educ. 1996;21:93–108. doi: 10.1080/03075079612331381487.

[4] Singh A , Ferry D , Mills S. Improving biomedical engineering education through continuity in adaptive, experiential, and interdisciplinary learning environments. J Biomech Eng. 2018;140:0810091–0810098. doi: 10.1115/1.4040359. 30003258 PMC6056190

[5] Beaudoin DE , Richardson SJ , Sheng X , Mitchell JA. Medical students’ perspectives on biomedical informatics learning objectives. Int J Med Educ. 2013;4:1–8. doi: 10.5116/ijme.50ce.316b.

[6] Borgatti SP , Halgin DS. Elicitation techniques for cultural domain analysis. In: The Ethnographer’s Toolkit. Vol 3. Altamira Press Walnut Creek, 1999: 115–151. (https://www.researchgate.net/profile/Stephen-Borgatti/publication/230786753_Elicitation_Techniques_for_Cultural_Domain_Analysis/links/5743093e08ae9ace8418bef1/Elicitation-Techniques-for-Cultural-Domain-Analysis.pdf). Accessed February 19, 2024.

[7] Ackley C , Rodriguez DG , Villa G. I didn’t notice that you were watching me”: exploring a user acceptance study to conduct cultural domain analysis online during the COVID-19 pandemic. Int J Qual Methods. 2023;22:16094069231164602. doi: 10.1177/16094069231164602. 37122441 PMC10116222

[8] Smith JJ , Borgatti SP. Salience counts—And so does accuracy: correcting and updating a measure for free-list-item salience. J Linguist Anthropol. 1997;7:208–209. doi: 10.1525/jlin.1997.7.2.208.

[9] Dengah HJF , Snodgrass JG , Polzer ER , Nixon WC. Cultural domain analysis: free lists. In: Systematic Methods for Analyzing Culture. Routledge, 2020: 15–26.

[10] Jain S , Pei L , Spraggins JM , et al. Advances and prospects for the human bioMolecular Atlas program (HuBMAP). Nat Cell Biol. 2023;25:1089–1100. doi: 10.1038/s41556-023-01194-w. 37468756 PMC10681365

[11] Border S , Ferreira RM , Lucarelli N , et al. FUSION: a web-based application for in-depth exploration of multi-omics data with brightfield histology. bioRxiv [Preprint]. 2024. doi: 10.1101/2024.07.09.602778. PMC12462499 40998789

[12] Border S , Lucarelli N , Eadon MT , El-Achkar TM , Jain S , Sarder P. Computational pathology fusing spatial technologies. Clin J Am Soc Nephrol. 2023;18:675–677. doi: 10.2215/CJN.0000000000000146. 36913267 PMC10278855

[13] Gravlee CC , Maxwell CR , Jacobsohn A , Bernard HR. Mode effects in cultural domain analysis: comparing pile sort data collected via internet versus face-to-face interviews. Int J Soc Res Methodol. 2018;21:165–176. doi: 10.1080/13645579.2017.1341187.

[14] Albughayl L , Beckford W. Cultural domain analysis. In: Okoko JM , Tunison S , Walker KD , eds. Varieties of Qualitative Research Methods: Selected Contextual Perspectives. Springer Texts in Education. Springer International Publishing, 2023: 109–114. doi: 10.1007/978-3-031-04394-9_18.

[15] Dengah II HJF , Brewis A , Johnson JC et al. The benefits of using cultural domain analysis in applied anthropology. Pract Anthropol. 2024;46:97–99. doi: 10.1080/08884552.2024.2345806.

[16] CMILab UF. Introducing FUSION. 2023. (https://www.youtube.com/watch?v=d1tHayLENEE) Accessed September 16, 2024.

[17] University of Florida Research Foundation, Inc. FUSION. 2024. (http://fusion.hubmapconsortium.org/) Accessed September 16, 2024.

[18] Owen WF. Interpretive themes in relational communication. Q J Speech. 1984;70:274–287. doi: 10.1080/00335638409383697.

[19] Wencelius J , Garine E , Raimond C. FLARES. 2017. (www.anthrocogs.com/shiny/flares/) Accessed February 10, 2024.

[20] Sutrop U. List task and a cognitive salience index. Field Method. 2001;13:263–276. doi: 10.1177/1525822X0101300303.

[21] Guze PA. Using technology to meet the challenges of medical education. Trans Am Clin Climatol Assoc. 2015;126: 260–270. 26330687 PMC4530721

[22] Eiland LS , Todd TJ. Considerations when incorporating technology into classroom and experiential teaching. J Pediatr Pharmacol Ther JPPT. 2019;24:270–275. doi: 10.5863/1551-6776-24.4.270. 31337989 PMC6633276

[23] Davies A , Hooley F , Eleftheriou I , Abdulhussein H , Davies AC. Applying co-design principles for the development of health education and workforce development. Stud Health Technol Inform. 2022;298:39–45. doi: 10.3233/SHTI220904. 36073453

[24] Romero V , Donaldson H. Human-centred design thinking and public health education: a scoping review. Health Promot J Aust Off J Aust Assoc Health Promot Prof. 2024;35:688–700. doi: 10.1002/hpja.802. 37643841

[25] O’Connor S , Zhang M , Trout KK , Snibsoer AK. Co-production in nursing and midwifery education: a systematic review of the literature. Nurse Educ Today. 2021;102:104900. doi: 10.1016/j.nedt.2021.104900. 33905899

[26] Corrigan O , Glynn M , McKenna A , Smeaton A , Smyth S. Student data: Data is knowledge – putting the knowledge back in the students’ hands. In: 2015. (https://www.semanticscholar.org/paper/Student-data%3A-data-is-knowledge-%E2%80%93-putting-the-back-Corrigan-Glynn/c2d51317914b27b03c10bfe1992be3ce6165a560) Accessed March 3, 2024.

[27] Nkomo LM , Daniel BK , Butson RJ. Synthesis of student engagement with digital technologies: a systematic review of the literature. Int J Educ Technol High Educ. 2021;18:34. doi: 10.1186/s41239-021-00270-1. 34778529 PMC8241468

[28] Zheng T , Xie H , Gao F , et al. Research and application of a teaching platform for combined spinal-epidural anesthesia based on virtual reality and haptic feedback technology. BMC Med Educ. 2023;23:794. doi: 10.1186/s12909-023-04758-4. 37880665 PMC10601272

[29] Favero TG. Using artificial intelligence platforms to support student learning in physiology. Adv Physiol Educ. 2024;48:193–199. doi: 10.1152/advan.00213.2023. 38269404

[30] Hu R , Fan KY , Pandey P , et al. Insights from teaching artificial intelligence to medical students in Canada. Commun Med. 2022;2:63. doi: 10.1038/s43856-022-00125-4. 35668847 PMC9166802

[31] Karabacak M , Ozkara BB , Margetis K , Wintermark M , Bisdas S. The advent of generative language models in medical education. JMIR Med Educ. 2023;9:e48163. doi: 10.2196/48163. 37279048 PMC10282912

[32] Lee H. The rise of chatGPT : exploring its potential in medical education. Anat Sci Educ. 2024;17:926–931. doi: 10.1002/ase.2270. 36916887

[33] Hare R , Ferguson S , Tang Y. Enhancing student experience and learning with iterative design in an intelligent educational game. British Br J Educ Technol. 2025; 56:551–568. 10.1111/bjet.13526.

[34] Border S. SarderLab/FUSION. (https://github.com/SarderLab/FUSION) Published online September 6, 2024. Accessed October 15, 2024.

