# Supplementary material for: Shaping learning objectives for biomedical artificial intelligence: Student-centered insights into novel cell visualization technology
Source: J Clin Transl Sci. 2025 Sep 26;9(1):e208. doi: 10.1017/cts.2025.10107 (PMC12505238; doi:10.1017/cts.2025.10107)

Appendix A1. Elicitation Interview Guide.

Q1: Can you tell me about a specific case where you find FUSION to be particularly useful?

Follow-up: For what other purposes do you think Fusion would be useful? What else?

Q2: When using FUSION, what features do you believe will be most important for the best user experience?

Follow up: What else would be relevant for other students like you? Can you think of any other features?

Q3: What aspects do you believe could be improved to enhance the usability of FUSION?

Follow-up: What other aspects do you believe could be further improved? Can you think of any other aspects?

Appendix A2. Data Saturation Plot.

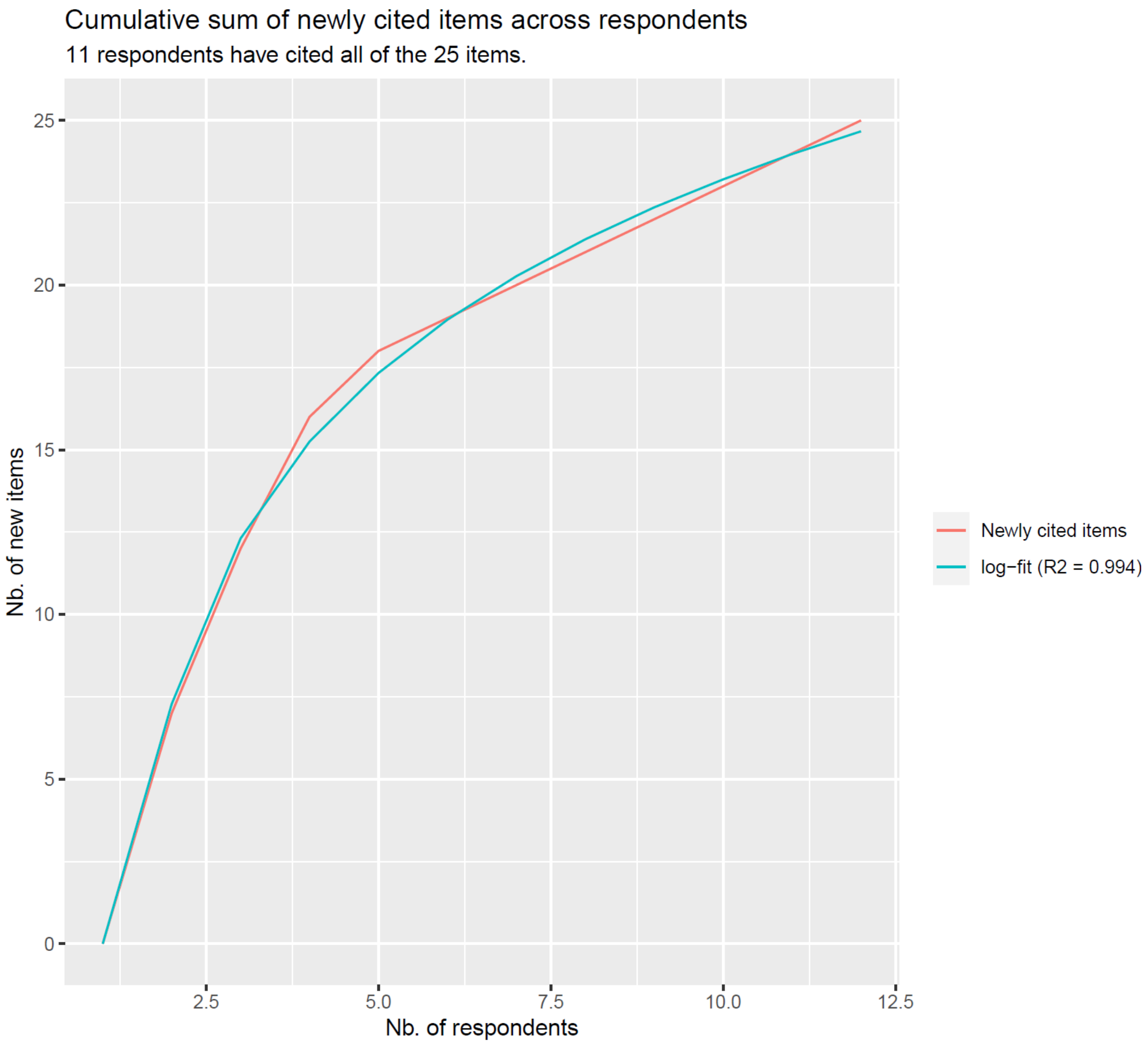

Supplement: Liu-Galvin et al. supplementary material [file S2059866125101076sup001.pdf]
